# Empirical and Bayesian approaches to fossil-only divergence times: A study across three reptile clades

**DOI:** 10.1371/journal.pone.0169885

**Published:** 2017-02-10

**Authors:** Alan H. Turner, Adam C. Pritchard, Nicholas J. Matzke

**Affiliations:** 1 Department of Anatomical Sciences, Stony Brook University, Stony Brook, New York, United States of America; 2 Department of Geology, Yale University, New Haven, Connecticut, United States of America; 3 Division of Ecology, Evolution, and Genetics, Research School of Biology, The Australian National University, Canberra, Australia; Museum für Naturkunde, GERMANY

## Abstract

Estimating divergence times on phylogenies is critical in paleontological and neontological studies. Chronostratigraphically-constrained fossils are the only direct evidence of absolute timing of species divergence. Strict temporal calibration of fossil-only phylogenies provides minimum divergence estimates, and various methods have been proposed to estimate divergences beyond these minimum values. We explore the utility of simultaneous estimation of tree topology and divergence times using BEAST tip-dating on datasets consisting only of fossils by using relaxed morphological clocks and birth-death tree priors that include serial sampling (BDSS) at a constant rate through time. We compare BEAST results to those from the traditional maximum parsimony (MP) and undated Bayesian inference (BI) methods. Three overlapping datasets were used that span 250 million years of archosauromorph evolution leading to crocodylians. The first dataset focuses on early Sauria (31 taxa, 240 chars.), the second on early Archosauria (76 taxa, 400 chars.) and the third on Crocodyliformes (101 taxa, 340 chars.). For each dataset three time-calibrated trees (timetrees) were calculated: a minimum-age timetree with node ages based on earliest occurrences in the fossil record; a ‘smoothed’ timetree using a range of time added to the root that is then averaged over zero-length internodes; and a tip-dated timetree. Comparisons within datasets show that the smoothed and tip-dated timetrees provide similar estimates. Only near the root node do BEAST estimates fall outside the smoothed timetree range. The BEAST model is not able to overcome limited sampling to correctly estimate divergences considerably older than sampled fossil occurrence dates. Conversely, the smoothed timetrees consistently provide node-ages far older than the strict dates or BEAST estimates for morphologically conservative sister-taxa when they sit on long ghost lineages. In this latter case, the relaxed-clock model appears to be correctly moderating the node-age estimate based on the limited morphological divergence. Topologies are generally similar across analyses, but BEAST trees for crocodyliforms differ when clades are deeply nested but contain very old taxa. It appears that the constant-rate sampling assumption of the BDSS tree prior influences topology inference by disfavoring long, unsampled branches.

## Introduction

Biologists and paleontologists need dated phylogenies to test a host of evolutionary questions ranging from global phenomena like climatic-biotic interactions through time and intercontinental historical biogeography, to more local or taxon-specific processes, such as estimating rates of morphological change, origination, and extinction. Fossils and the chronostratigraphic data associated with them are the only direct source of absolute timing for the Tree of Life. For neontological studies focused primarily on estimating dated phylogenies for extant taxa, the most common method for including absolute timing from fossils has been via prior probability distributions applied to internal nodes (node date calibrations). The problem of how to most objectively and effectively translate fossil specimens into node calibrations is difficult and has received treatment in general [1, 2], on specific issues such as selection of appropriate fossils [3, 4], and establishing best practices for fossil calibration choice and justification [5]. Methods to assess the quality of calibrations [6–9], and to account for the effects of calibration uncertainty on molecular dating have become increasingly common [10–13].

These advances are useful contributions to the scientific project of dating a tree of life. However, most of the tree of life is now extinct. Most extinct lineages do not have extant members from which genomic data can be collected, and their relationships can only be estimated from fossil morphological data. Dating these phylogenies is as important as dating trees of extant taxa for reconstructing the timetree of life.

Advances in node-calibration methods do not translate into advances in time-scaling fossil-only phylogenies. Node calibration methods have no analog in fossil-only trees, and it is non-contemporaneous fossil tips that possess the chronostratigraphic data necessary to directly time-scale the tree. Thus the question with fossil-only trees is how best to use these tip ages to inform the node ages of the tree.

**Fig 1:**
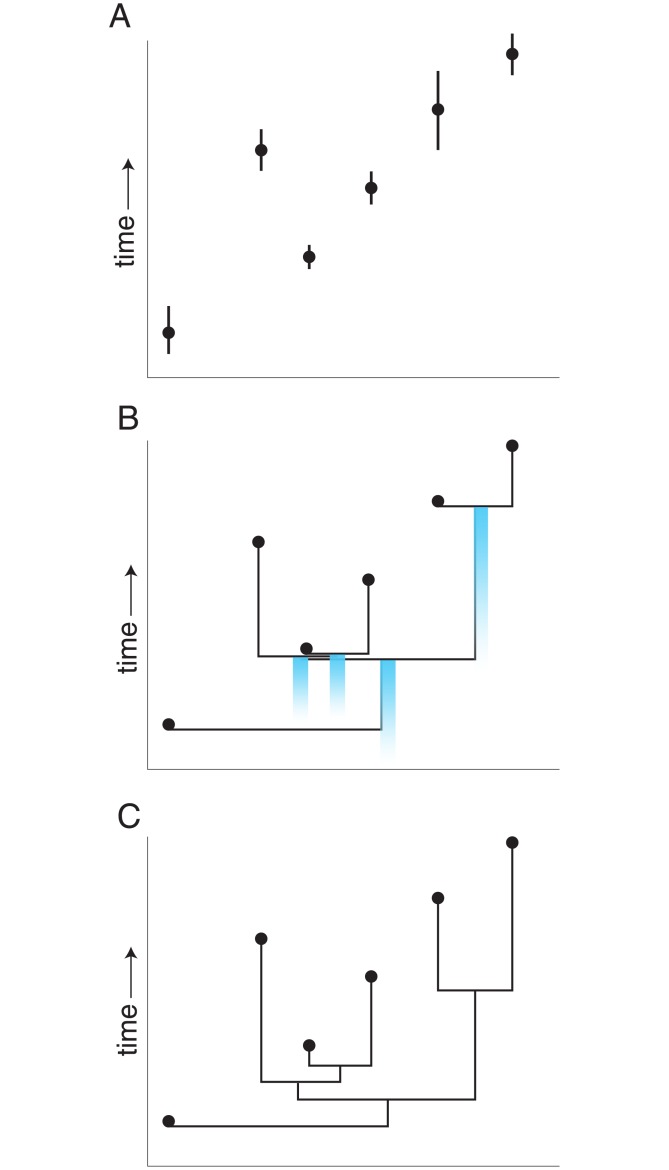
Time scaling a fossil phylogeny. **A**, Hypothetical temporal distribution of six fossil taxa; **B**, Strict temporal calibration of these taxa given a phylogeny resulting in inferred minimum estimate of divergence times between groups. Blue gradients denote the uncertainty associated with each calibrated node; **C**, Relaxed temporal calibration of these same taxa where the time separating two or more unconstrained nodes is divided evenly among those nodes and associated branches.

Any attempt to incorporate fossil data in timetrees should be cognizant of the various types of uncertainties inherent to the fossil record. Fossil tip ages have an associated uncertainty from to the stratigraphic uncertainty of the fossil age estimates [14] (Fig 1A). Moreover, because of varying preservation potentials, fossils likely underestimate lineage originations in the vast majority of cases [15]. The great challenge for fossil-only time calibration methods is balancing the uncertainty of the fossil tip ages with a metric to translate the absolute differences in those tip ages into a measure of branch length.

### Empirical approaches to dating phylogenies: Using paleontological data

Pre-phylogenetic approaches to dating the origins and durations of extinct species and taxa relied on a literal reading of first and last occurrence data from the fossil record [16–21]. The first attempts to produce dated phylogenies including fossils combined undated trees from cladistic parsimony analyses with the stratigraphic ranges of taxa. These time-calibrated cladograms relied on the assumption that sister lineages are reciprocally monophyletic and thus must have the same origination time. Any more remote relatives must branch earlier. Thus branching points are given minimum divergence dates based on the oldest member(s) of each sister group [22]. This can result in the prediction of unsampled fossil diversity (ghost lineages sensu Norell [22]). This approach to time-scaling a fossil phylogeny can be referred to generally as ghost lineage analysis (GLA) (Fig 1).

The occurrence of a fossil in the rock record represents temporal minimum for species or clade age [3, 15, 23]. A strict application of GLA returns only the minimum estimate of divergence times between groups and therefore reflects the minimum bound of the actual time of origin with the probability distribution of the actual time of origin remaining unknown (Fig 1B). Additionally, strict GLA may identify a number of temporally old taxa that are deeply nested in clades. These nested but old taxa provide the same minimum age constraint for several nested nodes simultaneously, thus resulting in some arbitrary number of zero-length internodes and terminal branches. Some paleontological studies utilize strict GLA to summarize empirical minimum clade origination dates (e.g., [24]) but for use in macroevolutionary analyses or phylogenetic comparative analyses strict GLA trees are not suitable. Zero-length branches are problematic both mathematically (they will crash many computer algorithms), theoretically (they are unlikely or impossible approximations of the true branch lengths), and biologically, as changes on these branches must be computed to occur at infinitely high rates.

Methods for time-scaling zero-length branches are needed to utilize GLA trees in comparative analyses. A common approach is to assign an arbitrary time-length to zero-length branches [25–30]. Alternatively, the time separating two or more unconstrained nodes can be divided evenly among those nodes and associated branches. This was originally described by Ruta et al. [31] and the division of time was scaled based on morphological divergence. Subsequent authors modified this approach and spread the time evenly across the unconstrained portion of the tree with no scaling metric used [27–30, 32, 33]. This general procedure can be referred to informally as “temporal smoothing” or “smoothed” GLA (sGLA) (Fig 1C).

### Model-based approaches to dating phylogenies: Using paleontological data

Using fossils to inform node-date calibrations remains, by far, the most common use of paleontological data in model-based dating procedures. This approach does not consider the wealth of other data fossils provide. Of particular importance is the fact that node-calibration does not allow fossils to inform the phylogenetic hypothesis, relying instead on ad hoc placement of fossils. Furthermore, as discussed above, it is not a viable time-scaling method for fossil-only datasets, or any dataset that contains extinct taxa lacking molecular data in the absence of a morphological model and unwarranted assumption of direct ancestry.

If the tips of a phylogeny are not all contemporaneous (as in fossil-only datasets), then an alternative to node-dating is available to calibrate the tree prior during divergence estimation. The variation in tip ages combined with estimates of evolutionary change (from either sequence or morphological characters) allow phylogenies to be calibrated to generate estimates of absolute rates and times. Tip-dating methods have been adapted to include fossil data and models of morphological evolution [9, 34] and are implemented in the programs BEAST [35–37] and MrBayes [38]. The use of tip-dating remains rare in combined fossil/extant datasets exploring evolutionary or biogeographic patterns but is increasing in popularity as the advocacy for “total evidence dating” increases [8, 39–44]. Furthermore, tip-dating has begun to be applied to morphology-based fossil-only datasets [45–48]. Tip-dating has advantages over the ad hoc methods of fossil node calibration because it fully integrates fossils and morphological/phenotypic data into the tree building process. Methodological advances and improved model choice apply equally to extant and fossil datasets. Insights from fossil-only datasets, such as variations in sampling through time and preservation potential, have the possibility to aid in improved tree priors from extant and combined datasets.

### Rationale

The choice of time scaling approach can have a strong effect on macroevolutionary and biogeographic analyses [24, 27, 28, 39]. In this paper, we explore empirically the utility of simultaneous estimation of tree topology and divergence times using BEAST tip-dating on datasets consisting only of fossils. We estimate timetrees using morphological datasets for three reptilian clades consisting exclusively or almost exclusively of extinct members. Two of the datasets were assembled by at least one of the authors and all are actively being maintained and expanded for analyses of phylogeny and paleobiology. We compare Bayesian and empirical time-scaling approaches. Although many researchers have focused attention on the reasonableness of applying relaxed clock models to morphological data, our main finding is that Bayesian tip-dating analyses may be more sensitive to violations of the tree prior than the clock-model, especially when sampling is strongly uneven through time.

## Methods

### Dataset choice

Three phenotypic datasets were selected that span the 270 million year history of Sauria, with a focus on basal saurians [49], basal archosaurs [50], and crocodyliforms [51]. The basal saurian dataset is derived from Pritchard et al. [49], which consists of 31 ingroup taxa and 246 phenotypic characters. The basal archosaur dataset is derived from Nesbitt [50], which consists of 76 ingroup taxa and 412 phenotypic characters. The crocodyliform dataset, derived from Turner [51], consists of 101 ingroup taxa and 318 phenotypic characters. Full dataset details are provided in Table 1, and all datasets are available to download on MorphoBank (www.morphobank.org).

**Table 1:** Dataset details.

Clade	# of Chars	# of Taxa	% Missing	Author	MorphoBank Project #
basal Sauria	246	31	31%	Pritchard et al., 2015	P854
basal Archosauria	412	76	37%	Nesbitt, 2011	P198
Crocodyliformes	318	101	39%	Turner, 2015	P1200

The datasets vary in both the amount of ingroup and character sampling. All datasets include a mixture of ordered and unordered characters and have a similar amount of missing entries (Table 1). These datasets overlap in the sampled higher taxa as well as some of the terminal taxa. Two are actively maintained by at least one of the authors, thereby providing a level of uniformity across datasets as to how the morphological characters are coded and scored. Other properties across these datasets include variation in taxonomic sampling density, different historical sampling intensities of their fossil records, as well as likely variable preservation potentials of the included taxa given the wide range in body size, habitat, and geographic provenance. Key divergences represented by the datasets include the lizard/bird split, the bird/crocodile split, the origin of crocodyliforms, and the major splits within crocodyliforms leading to the evolution of crown group Crocodylia.

### Tree estimation

Our choice of tree estimation procedure was motivated by our interest in comparing traditional empirical paleontological estimates to model-based tip-dating methods on fossil-only datasets. Phylogenetic relationships were estimated for each dataset using three different criteria; maximum parsimony (MP), undated Bayesian inference (BI), and simultaneous estimation of topology and divergence times in BEAST.

MP remains the most frequently employed tree building method for phenotypic datasets and its behavior is well known. MP trees were reconstructed using equally-weighted parsimony using TNT v1.5 [52–54]. A heuristic tree search strategy was conducted performing 10,000 replicates of Wagner trees (using random addition sequences, RAS) followed by TBR branch swapping (holding 10 trees per replicate). The best trees obtained at the end of the replicates were subjected to a final round of TBR branch swapping. Zero-length branches (i.e., no character change along the branch, not zero length in a temporal sense) were collapsed if they lacked support under any of the most parsimonious reconstructions (i.e., rule 1 of Coddington and Scharff [55]).

Bayesian inference (BI) trees were estimated using MrBayes v3.2 [56]. The standard model (Markov *k*-state variable model [M*k*v; [57]]) was specified for the phenotypic dataset with gamma-distributed rate variation (see Clarke and Middleton [58]). A subset of characters was set as ordered, following the prior usage of the included datasets. During the analysis, MCMC convergence was assessed using the average standard deviation of split frequencies and examining the trace files in Tracer [59]. Convergence to stationarity was assumed for split frequencies below 0.01 and ESS values >200. All analyses were performed with two runs of four chains each, run for 10 million generations, sampling parameters every 1000 generations. The first 25% of samples were discarded as burn-in. Results are summarized using maximum clade credibility trees. Of the four datasets examined only the crocodyliform dataset has been used to estimate BI trees [51, 60] and the results presented here match those of the previous analyses.

Tree topologies were estimated simultaneously with divergence times using a relaxed clock model implemented in BEAST 2 [37]. The details of this analysis are provided below under the “Tip-Dating” section. Dissimilar phylogenetic estimates from the same data would hinder comparisons between divergence dating methods like that undertaken here. Node ages can only be compared across methods if those nodes are shared between the estimated trees. By using all three procedures (MP, BI, BEAST), we will be able to distinguish the effects of the M*k*v model and the BDSS tree prior. Tip-dating in BEAST could provide different node ages from GLA if large deviations in estimated tree topology exist from more standard approaches.

### Ghost lineage analysis

The strict consensus MP tree and the maximum clade credibility BI tree obtained from each dataset were used to generate an empirically time-calibrated phylogeny using GLA. Two time-calibrated trees per dataset were generated corresponding to the strict GLA procedure and the “temporally smoothed” GLA procedure (sGLA). This was implemented using the R [61] software library *paleotree* [62]. Strict GLA trees correspond to the ‘basic’ option in the *paleotree* function TimePaleoPhy and the sGLA trees correspond to the ‘equal’ option. Under the ‘equal’ option, a value (vartime) must be provided that controls for the amount of time added to the root that will be using to smooth time along the unconstrained internodes. We chose three different values corresponding to 1 million years, 5 million years, and 10 million years. Ages for fossils occurrences are stratigraphic midpoints values or absolute values from radioisotopic dates if available (Supplementary Material).

### Tip-dating

We estimated mean node age and the 95% Bayesian credible interval (CI) using BEAST 2 [37]. Lineage-specific substitution rates were drawn assuming a 1-clock model with rates drawn from a single underlying uncorrelated lognormal distribution. Since all the phylogenetic data was in the form of phenotypic characters a single data partition was used and run under the M*k*v model [57] with a gamma-distributed rate variation. Characters were ordered as in the unconstrained MP and BI analysis outlined above. Tip dates were assigned a uniform prior distribution bounding the chronostratigraphic uncertainty of each terminal’s fossil occurrence. The distribution spanned the entire stratigraphic range for the fossil. The tree prior was set to a Birth-Death process with serial sampling (BDSS) [63]. This tree prior choice differs from early tip-dating analyses using fossil data. In Pyron [9] and Wood et al. [39] a Yule tree prior was used, although Wood et al. [39] also examined a Birth-Death tree prior but did not find results that differed from a strict Yule process. Exploratory use of Yule priors for our datasets resulted in extremely unrealistically old node estimates (Supplementary Material).

The NEXUS-formatted phylogenetic datasets and BEAST model run parameters were converted into BEAST’s unique XML format using *BEASTmasteR* [64, 65]. MCMC analyses were run in BEAST for twenty million generations, sampling the chain every 10000 generations. Log files were examined in Tracer [59] to confirm that ESS values for all parameters had reached 200 [66]. The first ten percent of samples were discarded as burn-in. The final chronogram and node ages were visualized in FigTree v1.4 (http://tree.bio.edu.ac.uk/software/figtree).

## Results

### Tree estimation

#### Sauria

**Fig 2:**
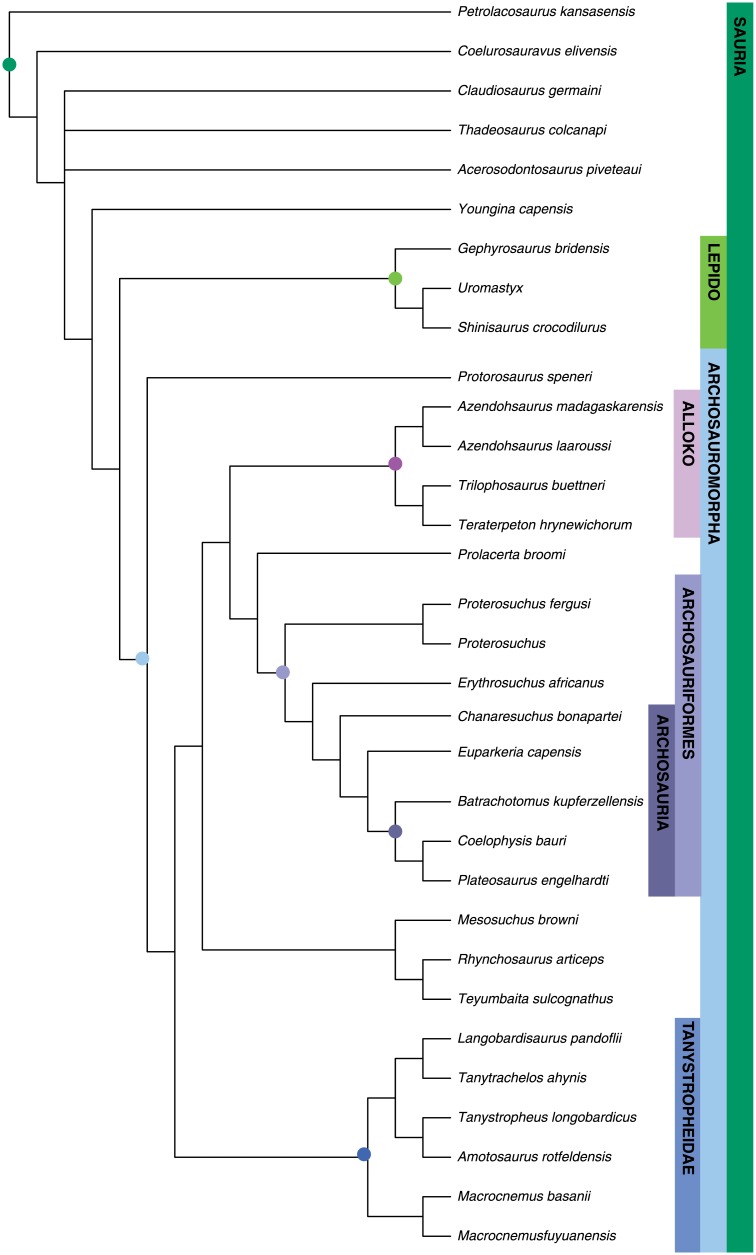
Maximum parsimony tree for Sauria dataset. Strict consensus of two most parsimonious trees (TL = 667, CI = 0.385; RI = 0.607). Major clades discussed in the main text are highlight for ease of comparison across figures.

Parsimony analysis resulted in two most parsimonious trees 667 steps long (CI = 0.385; RI = 0.607) (Fig 2). A monophyletic Archosauromorpha is recovered as the sister taxon to Lepidosauromorpha. *Protorosaurus* is recovered as the earliest-diverging archosauromorph. *Prolacerta* is recovered as the sister taxon to Archosauriformes, with Allokotosauria, Rhynchosauria, and Tanystropheidae being successively more distant sister taxa. Crown-group Archosauria is represented by *Batrachotomus*, *Coelophysis*, and *Plateosaurus*. The two most parsimonious trees differ in the placement of *Thadeosaurus* and *Acerosodontosaurus*, which are recovered as non-saurian diapsids in this analysis.

**Fig 3:**
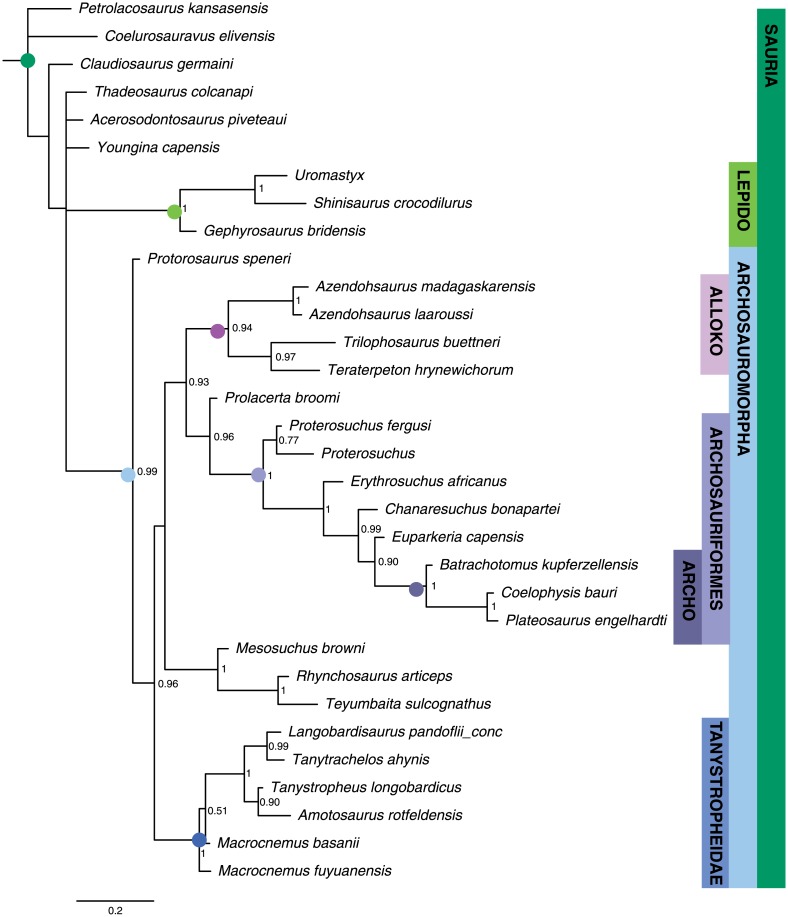
Bayesian inference tree for Sauria dataset. Maximum clade credibility tree with posterior probabilities displayed at the nodes. Major clades discussed in the main text are highlight for ease of comparison across figures.

The maximum clade credibility tree derived from the Bayesian analysis is well resolved except near the base (Fig 3). Support for most saurian clades is high (posterior probability (PP) > 0.90). Tree topology is largely congruent with Pritchard et al. and Nesbitt et al. [49, 67] and our MP analysis, with a monophyletic Archosauriformes, Tanystropheidae, Archosauromorpha, and Lepidosauromorpha all recovered with high posteriors. Poorly resolved basal relationships are likely the result of the highly fragmentary nature of some of the specimens/OTUs included for the temporal data provided by their old occurrences, and the limited character sampling for non-saurian diapsids in this dataset.

#### Basal archosauria

**Fig 4:**
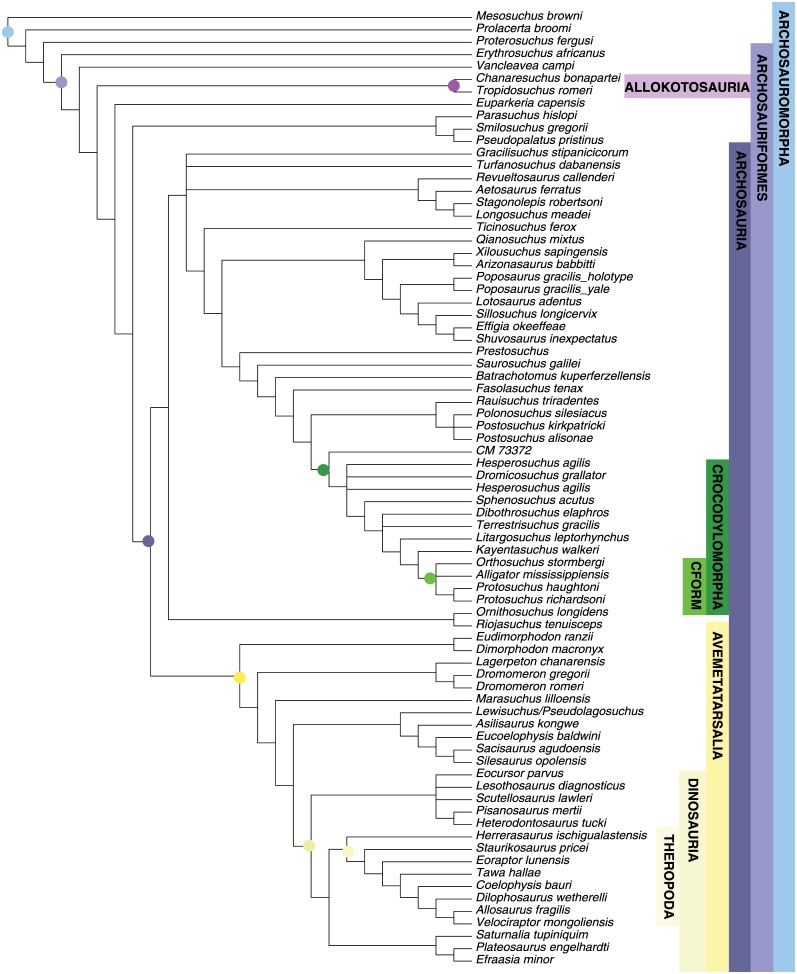
Maximum parsimony tree for Archosauromorph dataset. Strict consensus of 360 most parsimonious trees (TL = 1285, CI = 0.375; RI = 0.782). Major clades discussed in the main text are highlight for ease of comparison across figures.

MP analysis for the archosaur dataset resulted in 360 most parsimonious trees with a length of 1285 (CI = 0.375; RI = 0.782). The strict consensus tree (Fig 4) is identical to that recovered by Nesbitt [50], with the Phytosauria outside of Archosauria and a basal split in Archosauria between bird-line (Avemetatarsalia) and crocodile-line (Pseudosuchia) archosaurs. Avemetatarsalia includes dinosaurs and the more deeply nested theropods. Crocodylomorpha is monophyletic and Crocodyliformes is represented by *Protosuchus*, *Orthosuchus*, and *Alligator*.

**Fig 5:**
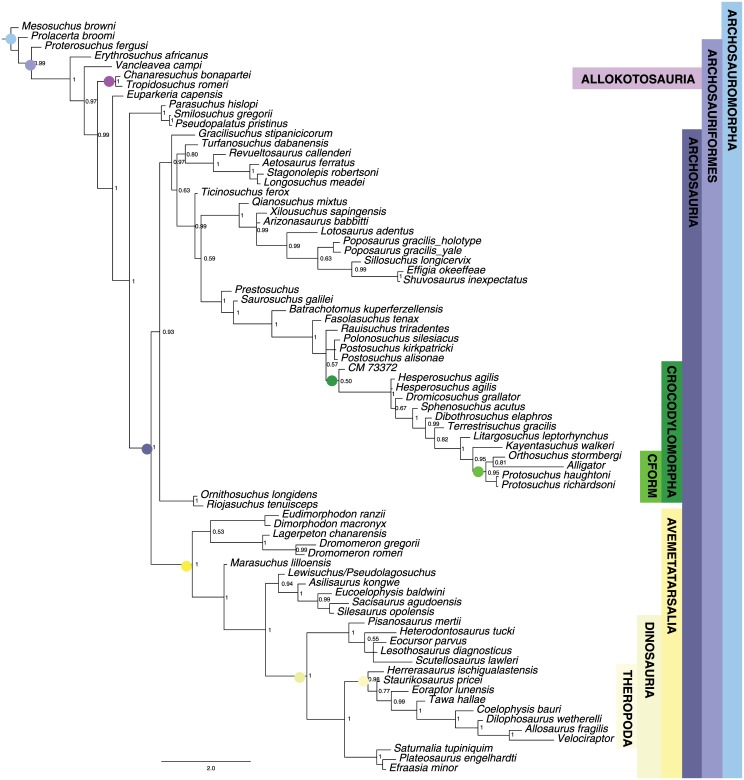
Bayesian inference tree for Archosauria dataset. Maximum clade credibility tree with posterior probabilities displayed at the nodes. Major clades discussed in the main text are highlight for ease of comparison across figures.

The maximum clade credibility tree from the Bayesian analysis is almost completely resolved and most nodes have posterior probabilities >0.90 (Fig 5). Ingroup relationships are highly similar to those recovered by Nesbitt [50] and our MP analysis.

#### Crocodyliformes

**Fig 6:**
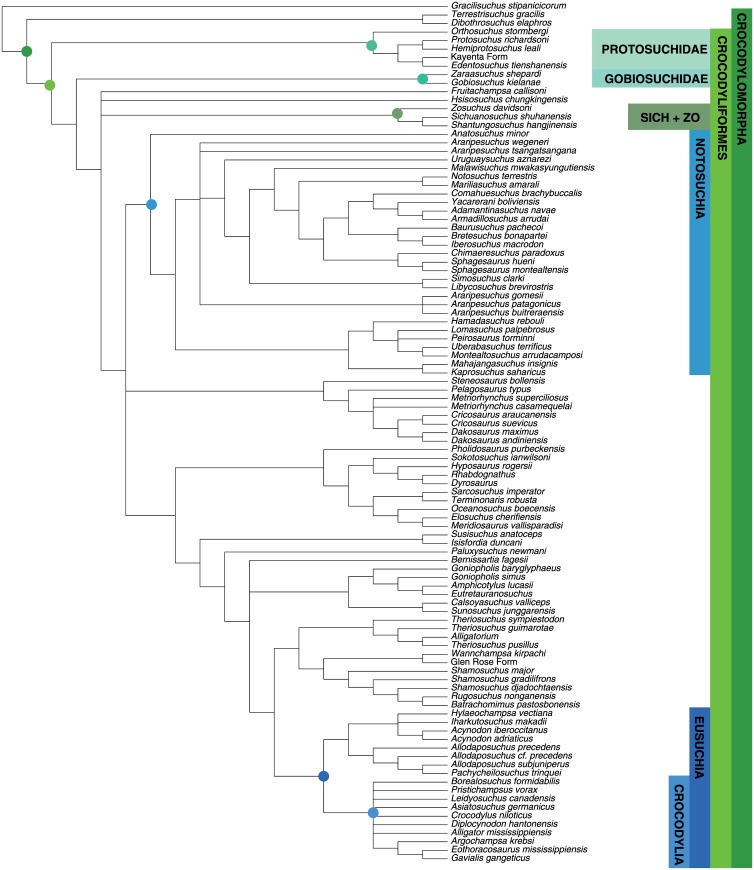
Maximum parsimony tree for Crocodyliformes dataset. Strict consensus of 108 most parsimonious trees (TL = 1662, CI = 0.239; RI = 0.700). Major clades discussed in the main text are highlight for ease of comparison across figures.

MP analysis of the crocodyliform dataset resulted in 108 optimal trees with a length of 1662 steps (CI = 0.239; RI = 0.700). A reduced strict consensus tree is presented in Fig 6. This consensus tree excludes the variable placement of *Bernissartia* in order to show the underlying tree structure shared among the most parsimonious trees. The results are the same as Turner [51] and recover the monophyly of most well established crocodyliform clades.

**Fig 7:**
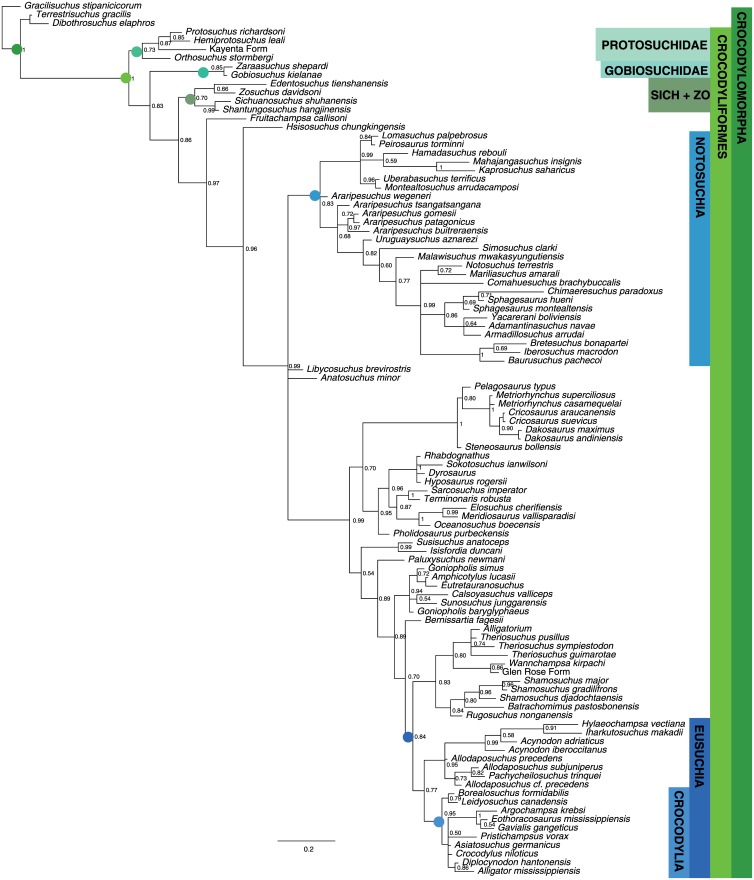
Bayesian inference tree for Crocodyliformes dataset. Maximum clade credibility tree with posterior probabilities displayed at the nodes. Major clades discussed in the main text are highlight for ease of comparison across figures.

Bayesian analysis resulted in a well-resolved maximum clade credibility tree depicting the monophyly of nearly all generally accepted higher-level clades (Fig 7) (see [68–72]). The clades Crocodyliformes, Thalattosuchia, Mesoeucrocodylia, and Neosuchia were all strongly supported (PP = 1.0), and a majority of nodes show posterior probabilities above 0.80. A monophyletic Notosuchia is recovered (PP = 0.83) but the notosuchians *Libycosuchus* and *Anatosuchus* are recovered in a polytomy at the base of Mesoeucrocodylia. This is likely due to the large amounts of missing data in these two taxa, which can result in BI pulling the incomplete taxa to the middle of the tree [58, 73]. A monophyletic Hylaeochampsidae plus *Allodaposuchus* clade is recovered as the sister taxon to Crocodylia, with Paralligatoridae (sensu Turner [51]) plus Atoposauridae as the next successive sister taxon.

### Ghost lineage analysis

Because of the overall topological similarity between trees estimated from BI and MP, we present ghost lineage analysis (GLA) results here using only the BI topologies. Selection of 1, 5, or 10 million years for the vartime value for sGLA had little to no effect on reconstructed node ages (Supplementary Material), as such we present only vartime = 1 results. The MP trees are available in the supplementary material.

**Fig 8:**
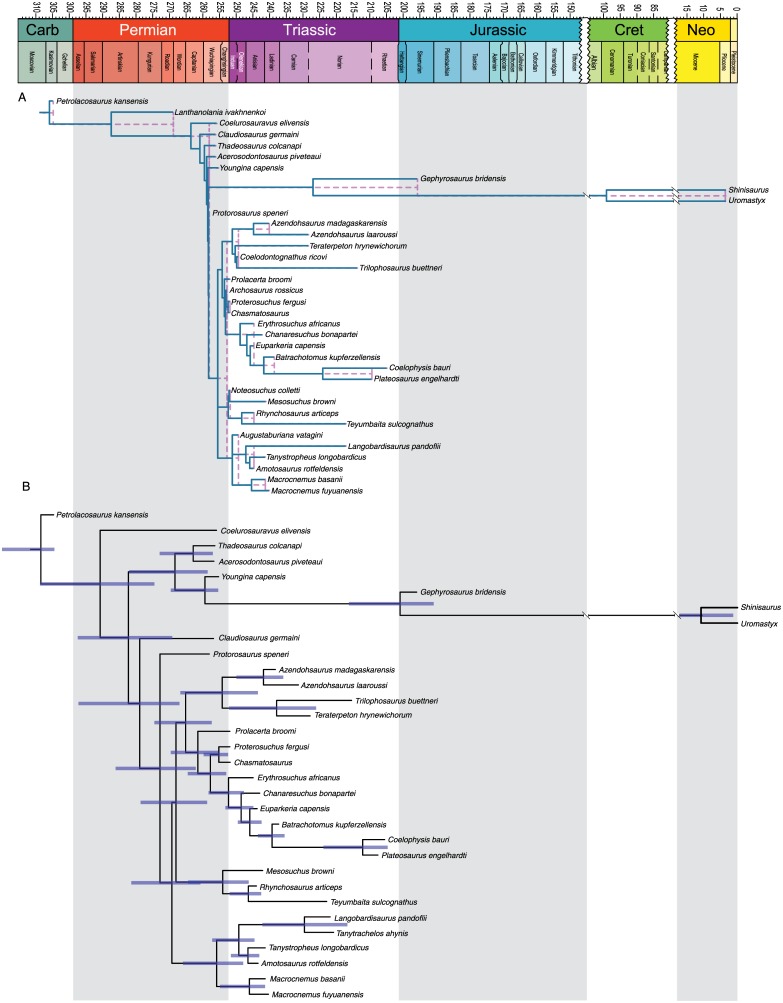
Ghost linage and BEAST2 analysis of Sauria dataset. A. Chronogram and minimum node ages for the Sauria dataset (BI results) obtained from strict GLA (light purple dashed lines) superimposed on the sGLA chronogram (solid dark blue lines). B. Chronogram based on the maximum clade credibility tree for the saurian dataset with branch lengths drawn to reflect BEAST divergence time estimations. Error bars reflect the 95% highest probability density. All trees scaled to geologic time scale above.

**Fig 9:**
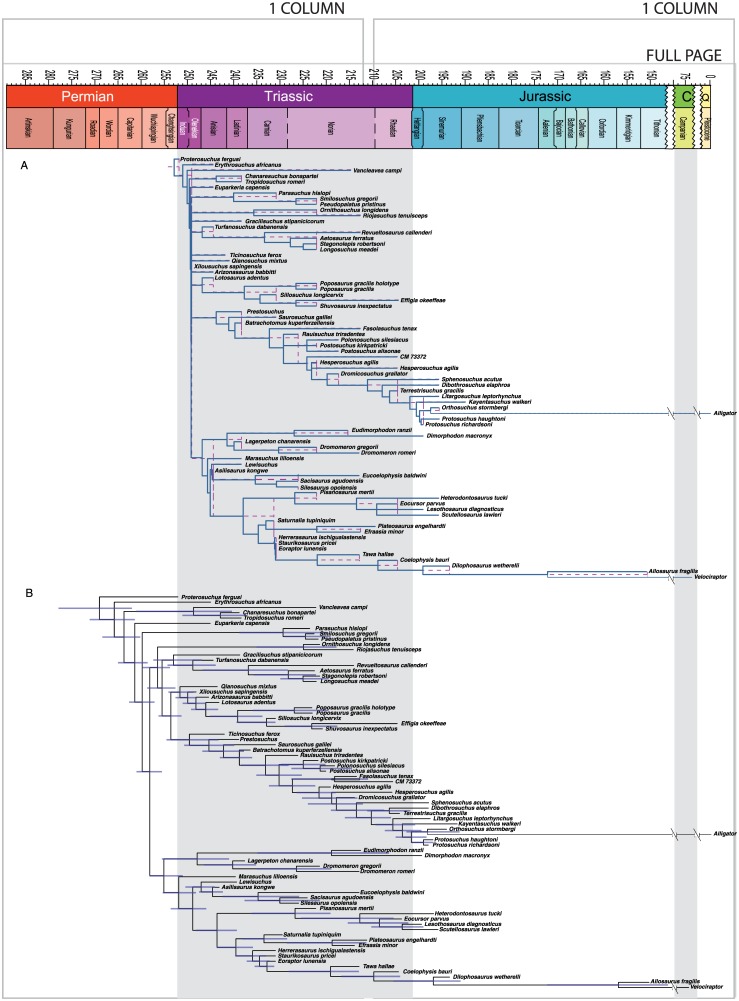
Ghost linage and BEAST2 analysis of Archosauria dataset. A. Chronogram and minimum node ages for the Archosauria dataset (BI results) obtained from strict GLA (light purple dashed lines) superimposed on the sGLA chronogram (solid dark blue lines). B. Chronogram based on the maximum clade credibility tree for the archosaur dataset with branch lengths drawn to reflect BEAST divergence time estimations. Error bars reflect the 95% highest probability density. All trees scaled to geologic time scale above.

**Fig 10:**
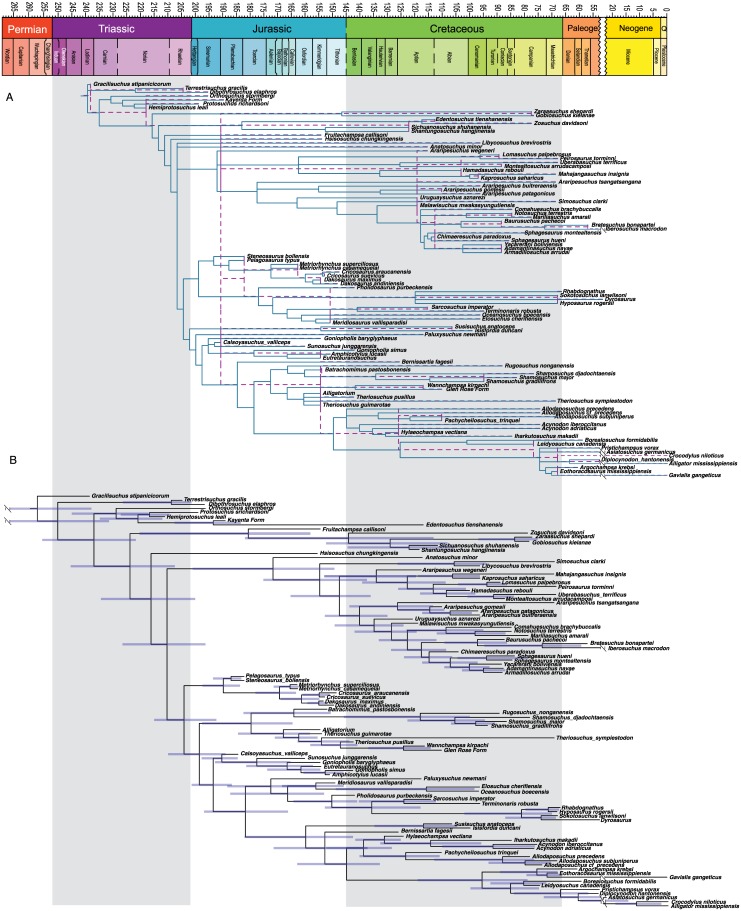
Ghost linage and BEAST2 analysis of Crocodyliformes dataset. A. Chronogram and minimum node ages for the Crocodyliformes dataset (BI results) obtained from strict GLA (light purple dashed lines) superimposed on the sGLA chronogram (solid dark blue lines). B. Chronogram based on the maximum clade credibility tree for the crocodyliform dataset with branch lengths drawn to reflect BEAST divergence time estimations. Error bars reflect the 95% highest probability density. All trees scaled to geologic time scale above.

Fig 8A shows chronogram and minimum node ages for the Sauria dataset obtained from strict GLA superimposed on the sGLA chronogram. Most nodes show only modest corrections using the sGLA approach. This is because most of the saurian taxa examined have occurrences clustered in a roughly 20 million-year time period spanning the Permo-Triassic boundary. The tight clustering of occurrences means most of the internodes are short branches and thus there is not much ‘time’ for the sGLA algorithm to spread across the nodes. Figs 9A and 10A show the same juxtaposition of strict GLA compared to sGLA results for the archosaur dataset and the crocodyliform dataset.

The archosaur nodes ages show a similar pattern to those of the saurian dataset. Much of the basal divergences in Archosauria are constrained by multiple taxa occurring in small time window in the Early Triassic. Occurrences among the crocodylomorphs and avemetatarsalians are less dominated by a single occurrence of old, deeply nested, taxa and therefore show larger changes in estimated node ages.

### BEAST analysis

#### Sauria

The maximum clade credibility tree derived from the BEAST analysis is very well resolved and similar to the BI and MP topologies in most respects (Fig 9B). Allokotosauria is closer to Archosauria than Tanystropheidae as in the BI and MP analyses. In the BEAST tree, *Youngina* and the *Acerosodontosaurus* + *Thadeosaurus* clade are on the Lepidosauria line as opposed to outside of the lepidosaur/archosaur split as in the BI and MP topologies. However, the posteriors on these relationships are very low. The basal relationships in the BEAST analysis are consistent with the BI tree, with *Coelurosauravus* as sister to Sauria, but with *Claudiosaurus* on the archosaur line as opposed to outside Sauria.

#### Archosauria

The maximum clade credibility tree is well resolved and similar to the MP and BI topologies (Fig 10B). The relationships are nearly identical to the BI tree, differing only in the placement of *Gracilisuchus*, *Turfanosuchus*, *Ticinosuchus*, and the “rauisuchian” taxa *Fasolasuchus* and *Rauisuchus*. Clade posteriors are similarly comparable to those estimated in the BI tree. The BEAST tree depicts a sister group relationship between *Turfanosuchus* and *Ticinosuchus*. *Gracilisuchus* is the sister taxon to *Revueltosaurus* + Aetosauria, whereas in the BI tree it is outside the Aetosauria/Crocodylomorpha split and in the MP tree its position is unresolved near this node.

#### Crocodyliformes

Among the three reptile datasets examined, the maximum clade credibility tree for Crocodyliformes deviates the most from the topology recovered by BI and MP. Major crocodyliform clades are recovered with high posterior probabilities, including Protosuchidae, Thalattosuchia, Mesoeucrocodylia, Notosuchia, Neosuchia, and Crocodylia (Fig 10B). The BEAST topology does not break up any well supported MP or BI groupings. However, three large neosuchian clades occupy widely different positions within Neosuchia compared to the BI trees. This includes Paralligatoridae and Atoposauridae [51] moving far to the base of Neosuchia, Tethysuchia moving uptree away from the thalattosuchians, and Susisuchidae moving from a basal position to a much more derived position. Ingroup relationships among atoposaurids and hylaeochampsid are largely unresolved in the BI tree, but BEAST resolves these clades to be temporally sequential. Neosuchia phylogenetic relationships remain in flux [74] and the exact nature of the differences between the MP, BI, and BEAST topologies are outside the scope of this paper. However, that the BEAST analysis does differ is important and will be discussed further below.

#### Divergence times

**Fig 11:**
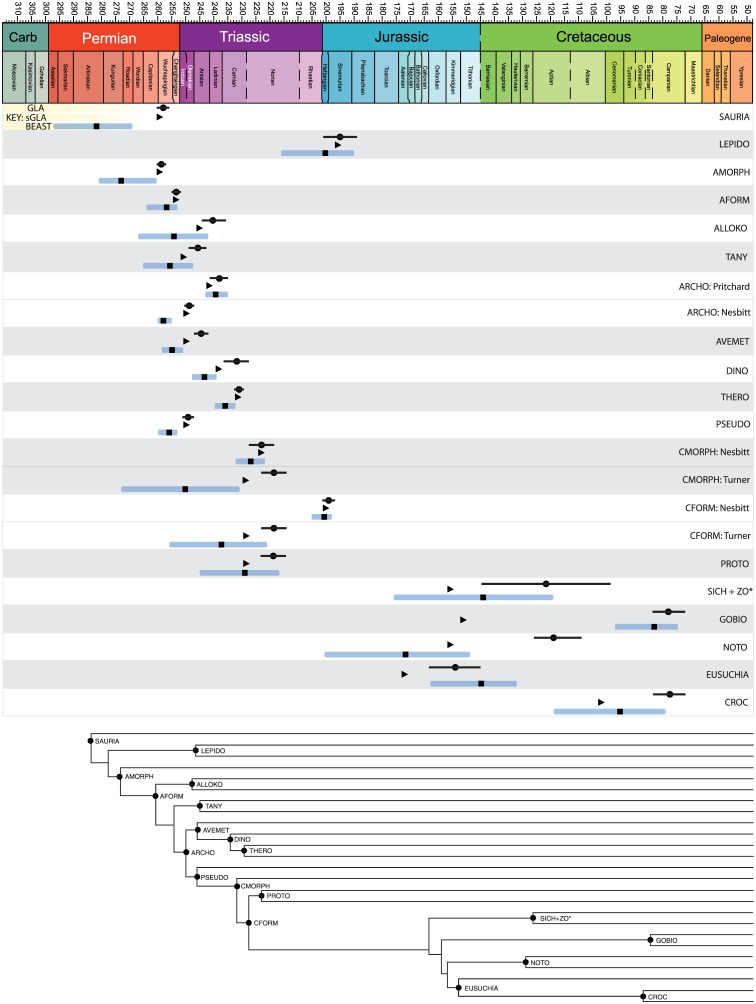
Node estimates compared across the GLA, sGLA, and BEAST methods. Nineteen key nodes were chosen for direct comparison across the three methods examined. Time scaled to geologic time scale above. Clade name abbreviations are on the right. For each clade name, the depicted node estimate from top to bottom go as follows; GLA estimate with solid bar showing the minimum to maximum stratigraphic error, sGLA estimate, BEAST estimate with error bar representing the 95% highest probability density. Key for these node labels is in the upper left hand corner. A summary cladogram is provided at the bottom.

Tip-dated node ages are presented in Figs 8–10 and available in the supplemental tree files (Supplementary Material). We chose nineteen key nodes representing major saurian clades present across the three datasets in order to compare the estimates from GLA, sGLA, and BEAST tip-dating (Fig 11). Three of these nodes are shared among at least two of the datasets. Table 2 summarizes the estimated node ages across methods.

**Table 2:** Comparison of node estimates from Fig 11.

Node	GLA	sGLA	BEAST2	Dataset
CROC	77.1	101.7	95.6	T15
EUSUCHIA	154.7	172.8	145.1	T15
NOTOSUCHIA	119.5	155.2	171.9	T15
GOBIOSUCHIDAE	77.9	134.4	83.7	T15
Sich + Zosuchus	122.5	156.8	144.3	T15
CROCODYLIFORMES	218.5	228.9	237.2	T15
CROCODYLIFORMES	199.2	199.9	201.7	N11
CROCODYLOMORPHA	218.5	239.5	250.4	T15
CROCODYLOMORPHA	222.3	223.3	227.3	N11
PSEUDOSUCHIA	249	249.3	256.3	N11
DINOSAURIA	231.5	238.1	243.6	N11
THEROPODA	231	231.3	235.9	N11
AVEMETATARSALIA	244.6	249	255	N11
ARCHOSAURIA	249	249.7	258.1	N11
ARCHOSAURIA	238.5	241.5	239.1	PEA15
TANYSTROPHIDAE	244.6	250.9	255.8	PEA15
ALLOKOTOSAURIA	240	245.9	254.1	PEA15
ARCHOSAURIFORMES	251.8	253	257.7	PEA15
ARCHOSAUROMORPHA	258	258.1	272.8	PEA15
LEPIDOSAUROMORPHA	195.6	226.8	200.7	PEA15
SAURIA	258	258.3	282.3	PEA15

## Discussion

### Bayesian inference of morphology returns similar tree topologies as parsimony

One possible hurdle to adoption of model-based divergence dating methods in fossil-only datasets is the need to use probabilistic models for phenotypic change. Parsimony remains the most commonly employed method to estimate trees using phenotype-only datasets and the use of probabilistic models for morphology has been questioned on both theoretical and practical grounds [73, 75]. Although the number of published analyses using likelihood or Bayesian inference with phenotypic datasets is increasing, there remains a paucity of data pertaining to whether estimated tree topologies differ considerably between the methods. Recent work by Wright and Hillis [76] and O’Reilly et al. [77] present a more positive view of Bayesian phylogenetics for morphology, as their respective simulations indicate stronger performance of the M*k*v model over parsimony.

For the three datasets we examined, the BI tree topologies differed only slightly from the MP trees. For the crocodylomorphs, 26 tips require moves to match the two topologies. Most of these moves are one internode moves or resolving polytomies. For the archosaur tree, moving 11 tips match the two trees, 9 of those are resolving polytomies and the remaining 3 are one node jumps. The saurian trees are even better matched; five tip moves match the topologies, with all but one resolving a polytomy. Although both MP and BI trees possessed polytomies, the BI trees for all datasets had more polytomies than the MP tree. This finding is inline with the recent simulation study of O’Reilly et al. [77] that found the M*k*-model recovers trees with less resolution than parsimony in analyses of morphological data.

This suggests that, at least for the datasets we have examined, the utilization of the M*k*v model does not dramatically alter the inferred phylogenetic relationships. This is an obviously useful result since it allows for comparisons between probabilistic methods and parsimony-based time calibration methods like GLA. It also provides a useful starting point to compare the effects of the additional model parameters that must be specified in BEAST tip-dating analysis. Deviations of the BEAST topology from the BI topology should provide a conservative indication of the effect that the tree and rate priors have on topology estimation, which in turn can influence divergence estimates for particular clades.

### “Smoothed” ghost lineage analysis mimics effects of the BDSS tree prior…sometimes

Results of divergence estimation across methods proved to be dataset dependent, but some general patterns are present. GLA and sGLA returned similar node estimates for the saurian and archosaur datasets with the sGLA values typically within the stratigraphic error of the GLA estimates (Fig 11). The median sGLA “correction” to the GLA value (as expressed as the difference between the sGLA estimate and the GLA value) is approximately 3 million years for both the saurian and archosaurian trees (Table 3).

**Table 3:** Average node “Correction” (in millions of years).

Dataset		sGLA correction	BEAST2 correction	% correction difference (BEAST2/sGLA)
Sauria	mean	10.8	7.6	70%
	median	3.3	7.6	230%
Crocodyliformes	mean	13.8	11.5	80%
	median	9.8	10.3	100%
Archosauria	mean	3.7	5.0	130%
	median	2.8	4.3	150%

This relationship did not hold for the crocodyliform dataset, where sGLA estimates tended to be considerably older than the GLA values and, at least for the nodes of interest, the sGLA lie outside the range of stratigraphic uncertainty for the oldest fossils establishing the GLA estimate (Fig 11). The median sGLA “correction” to the GLA values for the crocodylomorphs was nearly 10 million years.

The BEAST estimates are generally older than the sGLA estimates, but the 95% highest probability density (HPD) typically overlaps with the sGLA estimate (Fig 11). Nevertheless the saurian and archosaur datasets showed proportionally larger median node “corrections” (as expressed as the difference between the BEAST estimate and the GLA value) than the crocodyliform dataset. This is best shown in the percent correction of BEAST over sGLA values. For the saurian dataset the median node “correction” of the BEAST estimate was over two times greater than the sGLA value and for the archosaur dataset the BEAST estimate was one and half times greater than the sGLA value. The BEAST estimates for the crocodylomorph tree were most similar to the sGLA estimates, with a median node “correction” roughly equal to sGLA. This is consistent with the fact that the crocodyliform dataset yielded overall older sGLA estimates than the other two datasets.

The most notable deviation from the pattern of slightly older BEAST nodes estimates are with clades that consist of morphologically similar species that sit on long unsampled ghost lineages (see Gobiosuchidae node in Fig 9; GOBIO in Fig 11). For the saurian dataset, 6 of the 20 nodes (30%) in common between the sGLA tree and the BEAST tree were estimated to be younger by BEAST than by the sGLA algorithm. For the archosaur dataset it was 42% of nodes (24 of 57), and for the crocodylomorphs it was 50% (17 of 34 nodes) of nodes shared between the two topologies that were estimated to have younger divergence dates by BEAST than by sGLA. The estimate for the Gobiosuchidae node in the crocodyliform dataset is perhaps the most notable of these younger BEAST estimated nodes. The sGLA estimate for Gobiosuchidae is considerably older than the GLA estimate (153.6 Ma v. 82.6 Ma). The BEAST estimate of this node is instead 83.7 Ma and the 95% HPD overlaps slightly with the stratigraphic uncertainty of the oldest gobiosuchid. Two gobiosuchids are present in the dataset and are from the same deposit (Early Campanian, Djadokhta Formation). These taxa sit on a 71 million year ghost lineage. The sGLA algorithm places their divergence at the midpoint of this unsampled lineage. However, the two gobiosuchids are extremely morphologically similar suggesting a more recent divergence from one another. Since the BEAST estimate is moderated by the estimated rate of morphological evolution it appears to be behaving favorably in estimating a more recent split between the two gobiosuchids.

### Dataset choice is an important driver of node age estimation

Because the three datasets we examined overlap taxonomically, we can see how the choice to data (both characters and taxa) affects divergence estimates. Fig 11 shows the three major reptile groups whose divergences are estimated in at least two of the three datasets. The divergence of Archosauria was estimated in the saurian dataset (sGLA: 241.5, BEAST: 239.1) and the archosaur dataset (sGLA: 249.7, BEAST: 258.1). Crocodylomorpha was estimated in the archosaur dataset (sGLA: 222.3, BEAST: 227.3) and in the crocodyliform dataset (sGLA: 229, BEAST: 250.4). Crocodyliformes was also estimated in the archosaur dataset (sGLA: 199.9, BEAST: 201.7) and in the crocodyliform dataset (sGLA: 228, BEAST: 237.2).

These node ages show the common pattern that when the node is deeply nested the sGLA and BEAST estimates do not differ much and the sGLA is included in the 95% HPD (Archosauria in the basal saurian dataset; Crocodylomorpha in the basal archosaur dataset; Crocodyliformes in the basal archosaur dataset). When the node is near the root of the tree, the BEAST estimates are much older than the sGLA and have very large 95% HPD. Thus it appears that sGLA and BEAST converge on a node age when there are a large number of nested taxa influencing the estimate so long as the estimated node is not near the root. It is difficult to know which method is underperforming. It could be that sGLA significantly underestimates node age because there are no long internodes to divide the divergence time on. Conversely, BEAST may be overestimating the node age because there are not enough taxa near the root to constrain the morphological rate. This points to the notion that taxon sampling within a dataset is a more important factor for node divergence estimation than is character sampling.

### Should tip-dating be favored over sGLA for fossil-only datasets?

For any given branch length “correction”, there is a linear relationship between the amounts of “correction” that sGLA provides and the length of the supporting branch, given that the method pulls nodes halfway down that supporting branch. Even for the zero length branches this relationship holds when one considers that the time of the previous non-zero supporting branch is being divided between the number of zero length internodes between it and the next non-zero length branch. Per internode, half of that allotted time is assigned to the internode and the other half to the adjacent pendent edge. Considered tree-wide sGLA “corrections” follow the hyperbolic paraboloid equation z = x/(y+1), where z = the amount of time added to the zero length branch, x = length of the prior non-zero supporting branch, and y = number of intervening zero-length internodes. Thus, short supporting branches still lead to shorter corrections and longer supporting branches lead to longer corrections. As a result of this procedure, sGLA is strictly sensitive to the distribution of sampled taxa in geologic time and the stratigraphic congruence of that sample. More precisely, the procedure is sensitive to how tightly clustered the occurrence data are in time.

**Fig 12:**
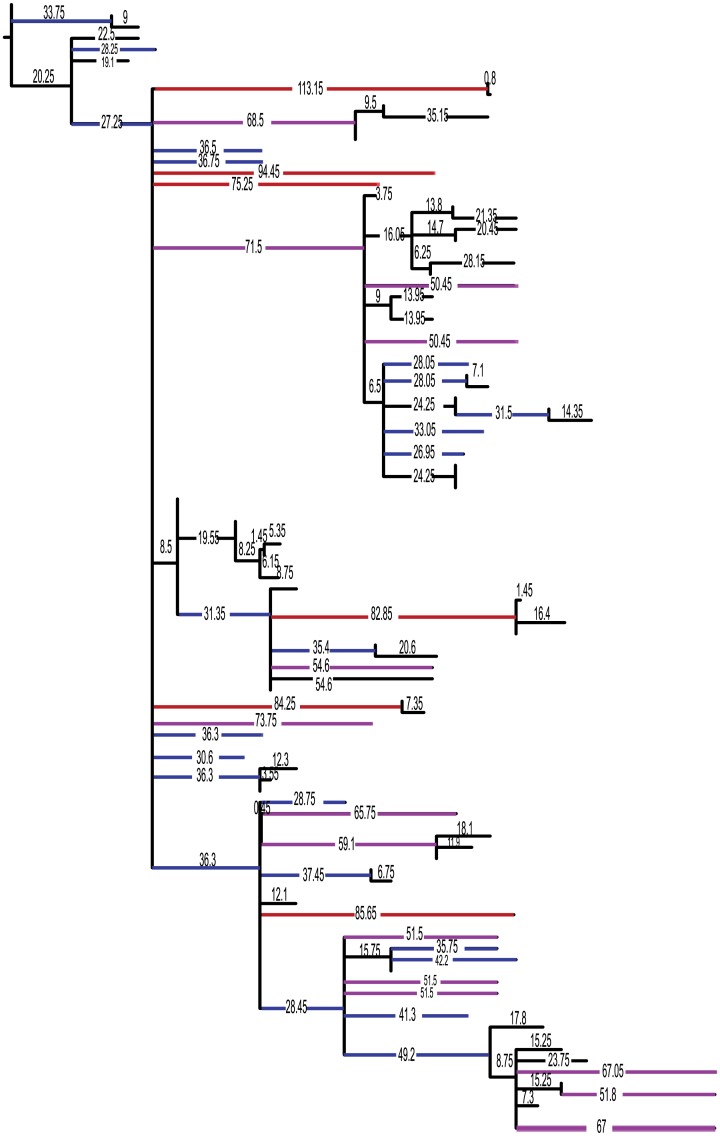
Ghost lineage lengths for Crocodyliform tree. Strict GLA chronogram of crocodyliform phylogeny illustrated the prevalence of extremely long unsampled lineages. All branches longer than 25 Ma have been highlighted. Blue branches are 25 Ma to 50 Ma, purple branches are 50 Ma to 75 Ma, and red branches of 75 Ma or longer.

This observation is borne out in the three reptile analyses we examined, which indeed show this to be a strong driver of node age correction. Both the saurian and archosaur datasets are more temporally evenly sampled tree-wide than the crocodyliform taxa. Their occurrences are more tightly clustered in time and proportionally more nodes are constrained by one old occurrence horizon. The crocodyliform dataset differs from this pattern with less tightly sampled species through time but with much less even sampling over the entirety of the tree (see average ghost lineage length in Fig 12), which is why the sGLA corrections are much larger for the crocodyliforms than for the other two datasets.

One potential criterion that would lead one to favor the tip-dating approach over sGLA would be if the method escaped the linear (hyperbolic tree-wide) node correction relationship. Another would be if it could ameliorate the effects of uneven temporal sampling of taxa. Tight temporal clustering of fossil occurrences often results in numerous basally diverging clades having their divergence estimate constrained by a single old occurrence point or by occurrence from a single old locality. This is evident in the three datasets we have analyzed. Basal archosauromorph nodes in the saurian dataset, basal archosauriform and archosaurian nodes in the basal archosaur dataset, and basal mesoeucrocodylian nodes in the crocodyliform dataset, all show the clear signs of minimal sGLA correction driven by a single old occurrence point (Figs 8 and 9). No such sensitivity to temporal constraints from a single old occurrence point or a single old fossil locality is apparent in the BEAST trees for any of the three datasets investigated (Figs 9 and 10). However, given the comparative approach of our study, we cannot say if the older BEAST estimates better reflect the true divergence times of these clades, or merely a reduced sensitivity to fossil occurrence heterogeneity. The BDSS prior we used assumes a constant rate of sampling through time (a newer “skyline” model allows birth, death, and sampling rate to change in different time bins, although rates will still be constant within time bins). Little is known regarding how robust tip-dating analyses are to violations of this assumption. In our empirical datasets, the saurian and basal archosaurs have generally fairly even sampling through time. The same is not true for the crocodyliform dataset, which shows a strongly heterogeneous sampling and the greatest disparity in topology between BEAST and BI/MP analyses. Sensitivity analyses wherein we excluded all extant taxa from the datasets, thereby further evening the sampling through time, does not change this general result (Supplementary Material).

### BDSS prior violations results in large topology deviations from MP or BI trees

As discussed above, Bayesian inference using the M*k*v model returns similar tree topologies as MP for the three dataset we examined. The notable exception to this was with the crocodylomorph dataset. The BEAST-estimated topology deviates from commonly recovered MP or BI crocodylomorph phylogeny. In particular, basal crocodyliform and neosuchian interrelationships show the biggest deviation from the MP and BI inferred trees. Thirty-eight tips required moves to match the BI tree to the BEAST tree. Whereas 13 of these moves were resolving polytomies, the remaining 25 were substantial moves spanning numerous nodes. The BEAST topology breaks up well supported neosuchian groups, unites most basal crocodyliforms into a single clade, moves basal neosuchian clades such as Susisuchidae to derived positions within Neosuchia, and moves clades that are often deeply nested in Neosuchia down into basally diverging positions within Crocodyliformes (e.g., Thalattosuchia, Hylaeochampsidae).

A chronogram for crocodylomorphs using GLA shows the striking number of very long ghost lineages for the group (Fig 12). Twenty-one taxa sit on unsampled lineages over 50 million years in duration, 11 of which are over 65 million years in duration. The average ghost lineage for the group as sampled is 31 million years. This level of uneven sampling strongly violates the BDSS tree prior assumption of constant sampling through time. It is clear from looking at the estimated BEAST topology that the most striking difference from the BI topology is that the BEAST tree greatly reduces ghost lineages by disfavoring long, unsampled branches.

## Conclusions

Tip-dating in BEAST generally performs well when compared to sGLA. Both recover reasonable divergence estimates for most nodes in densely sampled regions of the phylogeny. Tip-dating appears to be correctly moderating the node-age estimate (otherwise overestimated by sGLA) based on the limited morphological divergence of closely related taxa, sitting on long unsampled lineages. Tip-dating also is not constrained by the linear relationship between branch length and divergence “correction” that otherwise constrains the sGLA method. However, tip-dating provides very old node estimates near the root.

Long unsampled lineages confound both methods. In the crocodyliform dataset the sGLA values differ greatly from the GLA estimates and typically lay outside the stratigraphic error for the oldest occurrence driving the GLA estimate. Long unsampled lineages violate the constant-sampling-rate assumption of the BDSS tree prior and result in the estimate of tree topologies considerable different from the BI or MP trees.

These results suggest that when choosing a suitable method for estimated fossil-only node ages, researchers should take note of the potential biases in their data, such as unevenness of geologic sampling within their study taxa, and the prevalence of long unsampled lineages. These two factors will influence the results from a BEAST or sGLA analysis, perhaps more than choices about character data and relaxed clock models. In datasets with long unsampled lineages and a limited amount of character data, strong violation of the assumptions of the BDSS prior might make the sGLA approach a reasonable, more conservative option. In cases where sampling through time is more even, the BEAST approach has the advantage of non-linear node corrections and quantification of uncertainty in the form of 95% highest probability distributions.

The BDSS prior we have used here is a special case of a birth death skyline model (BDSKY—Stadler et al. [78]) that allows the sampling rate to change through time in a piecewise fashion. Future application of BDSKY models to fossil-only datasets is a promising avenue as it could model the uneven sampling of a particular extinct clade and potential avoid tree topology deviations like those exhibited in our tip-dating analysis of Crocodyliformes. On the other hand, skyline models have more parameters to estimate, so effective use of BDSKY models may require the consideration of informative priors on sampling rates in different time bins, perhaps incorporating knowledge from fossil databases or monographs about the quality of the fossil record in each time bin. However, although there is a massive literature on sampling, taphonomy, and bias in the fossil record, translating this into useful information for reasonable and coherent estimation of sampling rates for a set of specific OTUs coded in a morphological matrix may be a challenge. Multiple factors can influence fossil sampling rates ranging from preservation potential, to available outcrop area, to collection effort, and on top of this, other factors (specimen preparation, access, and completeness; OTU selection to answer specific questions in previous studies, etc.) will influence the sampling of OTUs in a particular morphological character matrix. More work is needed to further the use of these models and explore their efficacy.
